# The Metropolitan Asylums Board Report: 1914 a Strenuous Year

**Published:** 1915-09-18

**Authors:** 


### THE METROPOLITAN ASYLUMS BOARD REPORT.

### 1914 a Strenuous Year.

A strenuous year would be the appropriate description to apply to 1914 as viewed in retrospect through the report of the Metropolitan Asylums Board. Some part of the story of how the crowds of Belgian refugees were successfully dealt with by the officers of the Board has already been told in these columns, but the full account is now published in the recently issued seventeenth annual report. In addition to the enormous amount of extra work and organisation devoted to the thousands of refugees who passed through institutions managed by the Board, it must not be forgotten that the incidence of scarlet fever last autumn was unusually high, and in this direction also the resources of the Board were heavily taxed at about the same time. When it is borne in mind that mobilisation and enlistment made no small inroads among the staff just at the busiest season it will be freely admitted that the difficulties which had to be faced and were nobly overcome last autumn were of no mean order.

### The Ambulance Service.

It is not inappropriate to refer first of all to the ambulance service, for this is usually the point at which the public first comes in contact with the ever-widening network of the Board's activities. Last year was one of the most eventful in the history of the ambulance service; at a time when the ordinary work was greater than in any previous year the Board was called upon to give assistance in conveying sick and wounded sailors and soldiers sent for treatment in hospitals in and around London. The number of fever cases removed from their homes was 33,375, a total exceeding that of any previous year; in addition the number of private removals of infectious cases was more than double that of the previous year, and amounted to over 1,000. As may be imagined, the greatest difficulty was experienced in obtaining efficient drivers to replace those men who had joined H.M. Forces, and it is the more creditable to learn that the ambulance service record remains unbroken, and that no accident involving injury to any patient occurred during the year, notwithstanding that the journeys made reached the large total of 675,404 miles.

### The Casual Wards.

The comparatively new duties of the Board in connection with the casual wards of London are already familiar to our readers, but probably the great changes which have been made in the administration of these institutions are not yet fully appreciated. By means of a new chart in this report the results of the transfer of the casual wards to the Board's control are made very evident; the four coloured curves relating to the number of inmates on each Friday night during 1911, 1912, 1913, and 1914 demonstrate most vividly the very great reduction of casual pauperism that has taken place in the Metropolis. The Board took over twenty-four casual wards in April 1912, and at the end of last year only nine remained open. Of actual figures relating to the number of Friday inmates it must suffice to mention that in the four years referred to the highest was 1,170 in 1911, and the lowest only 164 in December last year.

Many of the items in the report of the Hospitals Committee have been recorded in our pages as they occurred. For example, the re-arrangement of appointments consequent on the resignation of Dr. Birdwood, the medical superintendent of the Park Hospital. Dr. Cameron, who was in charge of the Downs Sanatorium, is now superintendent of the river hospitals in place of Dr. Ricketts. At the Downs Sanatorium Dr. T. H. Woodfield is the acting medical superintendent.

### Fever Cases.

During the year the rise in the number of fever patients under treatment was so rapid that it became urgently necessary to suspend the admission of cases of measles and whooping-cough and also to circularise medical officers of health asking them to select for removal those cases of scarlet fever and diphtheria which most urgently required hospital treatment. The number of scarlet fever cases admitted to the hospitals was 22,006, a figure only once before exceeded—namely, in 1907, when there were 758 more cases; but last year the incidence of scarlet fever per 1,000 of the population of the Metropolis was higher than in 1907, owing to the fact that the census showed a decrease in the number of inhabitants in the area. The mortality rate for these cases was again very low—1.4 per cent., less than half of that ten years ago. In the case of diphtheria the admissions were absolutely higher than in any other year, and the death-rate was also greater—7.9 per cent. as compared with 6.2 in the two previous years. To accommodate these large numbers of patients several changes were made among institutions under the control of the Hospitals Committee. The Park Hospital reverted to the fever service in November, and the sick or debilitated children to whose use it has been devoted since 1910 were transferred to other institutions. The ringworm cases and those suffering from skin diseases were sent to the Goldie Leigh Homes, Abbey Wood, which were rented from the Woolwich Guardians. The Orchard Hospital, which was hurriedly erected in 1902 for smallpox purposes, but never occupied, was also used for the reception of fever patients. The Joyce Green Hospital, one of the river smallpox hospitals, was in use throughout the year as one of the regular fever hospitals. Preparations had been made in the middle of the year for the transference of the patients in the Fountain Asylum to an infirmary at Edmonton in order to free the institution for its original use for fever patients. Soon after the outbreak of the war the Edmonton Infirmary was utilised for the reception of Belgian refugees, and thus the proposal to return the Fountain Asylum to the fever service had to be suspended.

It is interesting to note that at the Brook Hospital the results obtained from the open-air treatment of patients on balconies provided by way of experiment to four of the wards were so successful that steps were taken to provide similar accommodation for open-air treatment outside all the other ward pavilions of that hospital. As has been often pointed out in these pages, this method of treatment is one which, judging by the favourable results obtained, especially in scarlet fever and measles cases, deserves to be utilised more extensively.

### Belgian Refugees.

The extent of the labours of those associated with the Local Government Board and the Metropolitan Asylums Board in the administration of refuges for the Belgians is hardly so well known as it deserves to be. A summary of the story is all that can be permitted here. On September 4 the Metropolitan Asylums Board was invited by the Local Government Board to undertake the provision and management of accommodation for war refugees arriving in London. On that day preliminary arrangements were made for opening the same evening the Crystal Palace, but on arriving at the Palace that afternoon the representatives of the Board found that they had been forestalled by the Admiralty a few hours before. In succession the old and dirty Poland Street workhouse was rendered habitable in less than twelve hours, and eventually occupied by Russian Jews; then the Hackney Wick refuge was converted on September 6, and subsequently used as an isolation hostel for families in which cases of infectious disease had occurred elsewhere; next the Edmonton workhouse was opened for the reception of 1,300 persons on September 7, and two days later 1,000 beds were available in St. Giles's Home, Endell Street.

The Alexandra Palace was taken over on September 11, and the admission of refugees began on the 14th. The work in this great building was described in an interview with Dr. H. Cuff, published in The Hospital for September 26, 1914. About 500 persons were admitted daily on an average, but on the occasion of the loss of the *Amiral Ganteaume* over 1,900 refugees were admitted after midnight. On October 12 St. Anne's Home, Streatham, was taken, and afforded accommodation for 500 people; and another institution in Edmonton, Millfield House, sheltered nearly the same number.

The Earl's Court Exhibition premises were taken over just as they stood on the morning after the last day of the exhibition, and by dint of extraordinary efforts the clearance of the general paraphernalia and the supply of equipment were so promptly proceeded with that by the same evening some 1,400 refugees were received between 9 and 11 P.M., and were fed and housed.

Lastly, temporary accommodation was provided for 800 Belgians at the Park Hospital during the interregnum while this institution was being reconverted to a fever hospital. In all, over 12,500 were provided, and by the close of the year 51,783 refugees were admitted; this number by the end of June 1915 rose to 94,005. Considering the difficulties which had to be contended with, especially those due to short staff and pressure of routine fever work, it must be admitted that the huge task was valiantly and creditably carried through.

